# Cognitive and emotional processing in brothers of individuals with anorexia nervosa: exploring familial cognitive vulnerability

**DOI:** 10.3389/fpsyt.2025.1641020

**Published:** 2025-08-29

**Authors:** Paolo Meneguzzo, Enrico Ceccato, Alessandra Sala, Paolo Santonastaso

**Affiliations:** ^1^ Department of Neuroscience, University of Padova, Padova, Italy; ^2^ Padova Neuroscience Center, University of Padova, Padova, Italy; ^3^ Vicenza Eating Disorders Center, Mental Health Department, Azienda ULSS8 “Berica”, Vicenza, Italy

**Keywords:** anorexia nervosa, empathy, emotions, siblings, alexithymia, theory of mind

## Abstract

**Background:**

Anorexia nervosa (AN) is associated with altered cognitive and emotional traits, including deficits in empathy, theory of mind (ToM), and increased alexithymia. While these traits are well-documented in affected individuals, little is known about their presence in unaffected male siblings, who share genetic and environmental risk factors. This study investigates whether male siblings of women with AN (bAN) exhibit intermediate cognitive-emotional traits compared to both their affected sisters and general population (GP) controls.

**Methods:**

We assessed 31 bAN, 31 GP, and 31 affected sisters. Participants completed self-report questionnaires (Empathy Quotient, Toronto Alexithymia Scale, Eating Disorder Examination Questionnaire) and two computerized tasks evaluating theory of mind (Story-based Empathy Task (SET), Reading the Mind in the Eyes Task). Between-group differences were assessed using non-parametric tests due to the non-normality of the data. A binary logistic regression was then conducted to evaluate whether specific socio-cognitive variables predicted group membership.

**Results:**

bAN showed significantly lower scores than the GP on measures of cognitive empathy and theory of mind, particularly Causal Inference (SET-CI; r = 0.594, p < 0.001), Emotional Attribution (SET-EA; r = 0.520, p < 0.001), and Intention Attribution (SET-IA; r = 0.463, p = 0.001). Logistic regression identified SET-CI as the strongest predictor of bAN status. Other empathy and alexithymia measures showed no significant group differences.

**Conclusion:**

These findings suggest that domain-general inferential difficulties—particularly in causal reasoning—may be associated with familial vulnerability to anorexia nervosa in male siblings. Further research is needed to clarify the role of such cognitive traits in the broader context of risk and to inform early identification and intervention strategies.

## Highlights

- Male siblings of AN patients show cognitive signs of social reasoning deficits.
- Causal inference may indicate familial vulnerability to anorexia nervosa.
- Screening at-risk male relatives could support early intervention strategies.
- CI-based tools may help detect subclinical cognitive traits linked to AN.
- Family-informed prevention models should consider social-cognitive profiles.

## Introduction

Anorexia nervosa (AN) is a severe and life-threatening eating disorder characterized by extreme food restriction, an intense fear of gaining weight, and distorted body image (1). AN predominantly affects individuals assigned female at birth, and it is considered one of the most gender-biased mental health disorders, although recent evidence suggests that prevalence in males may be underestimated due to underdiagnosis and diagnostic bias (2, 3). Understanding this gender disparity could provide valuable insights into how biological, psychological, and sociocultural factors interact in the development of psychiatric conditions. For instance, examining AN in the context of gender-related epidemiology may offer parallels with other conditions, such as autism spectrum disorder, where diagnostic biases and gender differences also play a significant role (4–6).

However, most research has focused almost exclusively on affected females, leaving the experiences and psychological characteristics of male family members largely unexplored. Investigating the impact of AN on male family members, such as brothers, could shed light on shared genetic and environmental risk factors, contributing to a more comprehensive understanding of the disorder (7). Given that brothers are less likely to develop AN themselves, but may still share predisposing factors, they offer a unique lens through which to examine whether certain traits associated with the disorder might exist independently of its clinical expression. In this sense, unaffected male siblings represent an ideal population for identifying trait-based markers that may signal familial liability, even in the absence of overt psychopathology.

Research has identified psychological traits such as empathy, theory of mind (ToM), and alexithymia as relevant to the understanding of anorexia nervosa beyond core eating symptoms, particularly due to their impact on interpersonal functioning, emotional regulation, and social cognition (8–10). Empathy refers to the ability to understand and share the emotions of others (11), while ToM involves the capacity to infer others’ mental states (12). Alexithymia, characterized by difficulty in identifying and describing emotions, has been frequently associated with AN, contributing to emotional dysregulation and impaired interpersonal relationships (13, 14). Gender differences in these traits are well-documented in the general population, with women generally exhibiting higher levels of empathy and TOM, while men are more prone to alexithymia (15, 16). This makes it essential to examine whether male siblings of individuals with AN, who are both genetically and environmentally linked yet phenotypically different, may show subclinical levels of these traits or express them differently. These differences raise important questions about how male and female siblings of individuals with AN might experience and express these traits.

While individuals with AN often exhibit deficits in empathy and ToM and elevated levels of alexithymia (17–19), it remains unclear whether these traits extend to their unaffected male siblings. Brothers of women with AN are an understudied group that could provide valuable insights into whether these psychological traits represent broader familial markers of vulnerability or are specific to the disorder (7). Moreover, recent research conducted during the COVID-19 pandemic has revealed that unaffected sisters of women with AN exhibited intermediate emotional response to stress, suggesting that siblings may experience subclinical levels of emotional dysregulation (20, 21). This finding raises important questions about whether brothers—despite being less frequently diagnosed with AN—might also display subtle emotional-cognitive differences, such as alterations in empathy, ToM, and alexithymia. If so, this could indicate that familial risk factors for AN extend beyond gender, potentially affecting male siblings as well. Similarly, the literature has already reported the presence of endophenotypic features in AN among unaffected sisters, such as self-shifting abilities and central coherence (22–24). Taken together, these findings support the hypothesis that cognitive-emotional traits related to AN may be heritable and expressed across the family system, even in individuals not affected by the disorder itself. Psychological traits such as empathy, ToM, and alexithymia may not only reflect core dimensions of AN psychopathology but may also serve as cognitive-affective endophenotypes—inheritable traits that mediate the pathway between genetic risk and clinical expression (25, 26).

### Aims and hypotheses

Building on these observations, the present study aimed to explore whether unaffected male siblings of women with AN display cognitive-emotional traits commonly associated with the disorder, even in the absence of clinical symptoms. We investigated empathy, theory of mind, and alexithymia in three distinct groups: brothers of women with AN (bAN), their affected sisters (AN), and men from the general population (GP).

Our primary hypothesis was that brothers of women with AN would show some degree of overlap in psychological traits with their affected sisters, reflecting shared familial vulnerabilities. Specifically, we hypothesized that (1) brothers would exhibit lower levels of empathy and theory of mind, and (2) higher levels of alexithymia compared to general population controls, although to a lesser extent than their sisters with AN.

By comparing these groups, we aimed to explore whether the psychological profiles of brothers align more closely with their affected sisters—indicating potential subclinical manifestations of traits associated with AN—or whether their profiles resemble those of men from the general population, suggesting more normative functioning. Understanding these dynamics is critical not only for identifying potential familial risk markers for AN but also for broadening our conceptualization of how cognitive-emotional vulnerabilities may manifest in family members who are not clinically affected. Ultimately, this approach could inform prevention strategies and improve our understanding of the transdiagnostic and transgenerational nature of emotional difficulties linked to eating disorders.

## Material and methods

### Participants

The main sample consisted of 31 male individuals who were the biological brothers of cisgender female patients diagnosed with anorexia nervosa (bAN). Female patients were recruited from the Eating Disorders Center of the ULSS 8 Berica in Vicenza, Italy. All women with AN were recruited through the specialized Eating Disorders Center of the ULSS 8 Berica Mental Health Department in Vicenza, a care unit providing both outpatient and inpatient treatment. Brothers were contacted during family involvement in treatment and assessment sessions, and each provided informed consent independently. In cases where multiple male siblings were eligible, the brother closest in age to the patient was selected to ensure demographic comparability.

The control group (GP) included 31 healthy male individuals from the general population, matched to the bAN group in terms of age and educational background. These participants were recruited through community advertisements and university outreach initiatives. All controls were screened to confirm the absence of any personal or familial history of eating disorders or other psychiatric conditions. They were all volunteers and were matched to the bAN group based on age and education.

Inclusion criteria for the AN group consisted of a formal diagnosis of anorexia nervosa according to DSM-5 criteria, regardless of subtype. Among the AN participants, 21 met criteria for the restrictive subtype and 10 for the binge-purging subtype. Male siblings were included if they were over 18 years of age, had no history of psychiatric diagnosis, and provided informed consent. For the control group, inclusion criteria mirrored those of the bAN group, with the added exclusion of any first-degree relatives with an eating disorder.

The study received ethical approval from the local Ethics Committee of Vicenza (approval code: VI-04/2021) and was conducted in accordance with the Declaration of Helsinki and international guidelines for research involving human participants. Written informed consent was obtained from all participants.

### Research protocol

All participants completed a battery of self-report measures, which included demographic information and standardized psychological questionnaires assessing empathy, theory of mind, alexithymia, and eating disorder symptoms. The first part collected self-reported data about age, gender, height, and weight. The second part included specific questionnaires, and a third part involved two computerized tasks.

Questionnaires were the eating disorder examination questionnaire (EDEQ), the empathy quotient (EQ) 15-item short form, and the 20-item Toronto alexithymia scale (TAS-20). The 2 computerized tasks were the story-based empathy task (SET) and the reading the mind in the eyes task (RMET).

The EDEQ is a self-report questionnaire that assesses the psychopathology specific to eating disorders (27). It provides a global score based on four subscales: Eating Concern, Weight Concern, Shape Concern, and Restraint. Scores reflect the severity of eating disorder symptoms. Participants are asked to rate the frequency of these behaviors over the last 28 days on a scale from 0 (never) to 6 (every day).

The 15-item short form EQ questionnaire is designed to measure empathy, assessing the ability to understand others’ emotions and adjust one’s behavior accordingly (28). The questionnaire contains 15 items, with responses scored on a four-point Likert scale. The EQ is divided into three subscales: Cognitive Empathy (EQ-CE), Emotional Empathy (EQ-EE), and Social Skills (EQ-SS). Higher scores indicate higher levels of empathy.

The TAS-20 measures alexithymia and is composed of 20 items divided into three subscales: Difficulty Identifying Feelings (TAS-DIF), Difficulty Describing Feelings (TAS-DDF), and Externally-Oriented Thinking (TAS-EOT; 29). Responses are scored on a five-point Likert scale, with higher scores indicating more severe alexithymia. A total score above 60 suggests high alexithymia, while scores of 51 or lower indicate no significant alexithymia.

The SET is a non-verbal task that assesses the ability to infer intentions and emotions through story vignettes (30). It includes 18 trials divided into three conditions: inferencing intentions (SET-IA), emotional states (SET-EA), and a control condition for causal inference (SET-CI). The total score (GSET) for the task is based on the number of correct responses, with a maximum score of 18.

The RMET assesses ToM by having participants identify emotions based on images of human eyes (31). The test consists of 36 photographs, each representing a different emotional expression, with participants selecting the correct emotion from four options. The final score is the number of correct responses, and the RMET has been widely used to measure emotion recognition in various populations.

### Statistical analysis

All statistical analyses were conducted using IBM SPSS Statistics, version 25. The distribution of continuous variables was assessed using the Shapiro–Wilk test. As several variables violated normality assumptions, non-parametric tests were consistently employed to ensure robustness across group comparisons. To evaluate differences across the three groups, we used the Kruskal–Wallis H test. This test was applied to both total and subscale scores of the relevant measures (i.e., SET, RMET, EQ, and TAS-20), regardless of statistical significance, to maintain transparency and reduce reporting bias.

For variables showing significant omnibus effects, *post hoc* pairwise comparisons were conducted using Bonferroni-corrected Mann–Whitney U tests. Given the familial link between individuals with AN and their brothers, paired comparisons between AN and bAN were also performed using the Wilcoxon signed-rank test. Effect sizes for non-parametric comparisons (r) were computed using Rosenthal’s formula (r = Z/√N).

A binary logistic regression was then conducted to assess whether specific cognitive-emotional variables—namely, subscales related to theory of mind (ToM), empathy, and alexithymia—predicted group membership (bAN vs. GP). Eight predictors were selected based on both theoretical relevance and significant between-group differences in preliminary analyses. We acknowledge that this number approaches the limits of model stability given our sample size (N = 62), and interpret findings accordingly as exploratory. To assess potential multicollinearity, Variance Inflation Factor (VIF) values were calculated for all predictors; all were below 3.0, indicating acceptable independence among variables. Additionally, a sensitivity analysis was conducted by removing the SET-CI variable from the model.

Sample size was determined based on recruitment feasibility and the matched design of the study. All available and eligible male siblings of individuals with AN treated at the clinical center during the recruitment window were included, in line with previous exploratory studies in this field (e.g., 7).

All tests were two-tailed, with a significance threshold set at p <.05.

## Results

Descriptive statistics are presented in **Table 1**, including paired comparisons between the bAN and AN groups, as well as between bAN and GP. Wilcoxon signed-rank tests showed that eating psychopathology differed significantly between bAN and their affected sisters. Additionally, specific differences in emotional features were observed across both comparisons. The bAN group exhibited lower scores on performance-based measures where lower scores indicate poorer outcomes (e.g., ToM and empathy tasks) and higher scores on self-report scales where elevated scores reflect greater emotional dysfunction (e.g., alexithymia subscales) when compared to the GP group. In contrast, the opposite pattern was generally observed when comparing bAN with their affected sisters.

**Table 1:** Demographic, clinical, and cognitive-emotional characteristics of participants in the anorexia nervosa (AN), brother (bAN), and general population control (GP) groups.

	bAN n = 31	AN n = 31	GP n = 31	bAN vs AN ^§^ Z (p)	bAN vs GP ^#^ Z(p)
Age, years	19.45 2.46	18.60 2.49	19.00 1.65	1.89 0.059	0.81 0.417
BMI, kg/m^2^	21.44 2.55	16.71 0.83	22.03 2.65	6.68 < 0.001 *	0.45 0.651
Years of education	12.61 1.91	11.97 1.60	12.42 0.92	1.87 0.061	0.13 0.892
EDEQ					
Restraint	0.66 1.16	2.55 1.65	0.52 1.01	4.14 < 0.001 *	1.34 0.180
Eating Concerns	0.53 1.03	3.25 1.69	0.23 0.35	4.75 < 0.001 *	0.74 0.459
Weight Concerns	0.97 1.17	3.93 1.72	0.73 0.72	4.69 < 0.001 *	0.29 0.773
Shape Concerns	1.35 1.44	4.19 1.64	1.05 0.95	4.60 < 0.001 *	0.63 0.527
Global Score	0.88 1.12	3.48 1.47	0.63 0.63	4.76 < 0.001 *	0.51 0.609
EQ					
EQ-CE	6.32 2.07	6.03 2.39	5.81 2.56	0.43 0.671	0.65 0.515
EQ-EE	6.13 1.84	7.16 1.85	6.00 1.75	2.01 0.045 *	0.22 0.823
EQ-SS	6.13 2.16	4.42 2.50	7.61 2.08	2.78 0.005 *	2.50 0.013 *
Total EQ	18.58 2.90	17.61 4.24	19.42 3.54	1.02 0.310	0.72 0.473
TAS-20					
TAS-DDF	15.06 4.58	18.16 5.44	12.61 3.68	1.92 0.055	2.48 0.013 *
TAS-DIF	17.42 6.09	25.39 6.77	13.77 6.09	3.91 < 0.001 *	2.81 0.005 *
TAS-EOT	19.84 4.12	18.29 5.22	17.48 3.80	0.94 0.346	2.46 0.014 *
Total TAS-20	52.32 11.14	61.84 14.76	43.87 9.07	2.00 0.046 *	3.06 0.002 *
RMET	24.00 2.32	22.97 2.68	25.45 1.82	3.28 0.001 *	2.07 0.039 *
SET					
SET-IA	5.10 0.79	4.74 0.89	5.48 0.72	1.81 0.071	2.16 0.031 *
SET-CI	4.65 0.61	4.42 1.18	5.45 0.62	0.81 0.419	4.34 < 0.001 *
SET-EA	4.97 0.66	4.48 0.93	5.45 0.57	2.32 0.020 *	2.87 0.004 *
GSET	13.65 1.76	14.71 1.57	16.39 1.36	2.11 0.035 *	4.15 < 0.001 *

Means and standard deviations are reported. Two independent comparisons were performed: bAN vs. AN (Wilcoxon signed-rank test #) and bAN vs. GP (Mann–Whitney U test §). EDEQ, eating disorder examination questionnaire; EQ, empathy quotient; CE, cognitive empathy; EE, emotional empathy; SS, social skills; TAS-20, Toronto alexithymia scale; DDF, difficulty describing feelings; DIF, difficulty identifying feeling; EOT, externally oriented thinking; RMET, Reading the Mind in the Eyes Test; SET, story-based empathy test; IA, identifying intentions; EA, emotional states; CI, causal inference; GSET, Global score of SET. *Denotes statistically significant differences (p <.05).

Notably, the bAN group significantly differed from the GP group on several scales: EQ-SS (r = 0.361), TAS-DIF (r = 0.363), TAS-EOT (r = 0.360), TAS-20 Total (r = 0.450), RMET (r = 0.302), SET-IA (r = 0.291), SET-CI (r = 0.594), and SET-EA (r = 0.381). Comparisons between bAN and AN also revealed differences in SET-EA (r = 0.314), SET-EE (r = 0.313), SET-SS (r = 0.388), TAS-DDF (r = 0.348), TAS-DIF (r = 0.613), TAS-20 Total (r = 0.422), and eating psychopathology (all r > 0.700).

To assess group differences across cognitive and emotional variables, Kruskal–Wallis H tests were conducted on total and subscale scores across the three groups. The analysis revealed significant omnibus effects for several key measures. Pairwise comparisons using Bonferroni correction showed that both the AN and bAN groups scored significantly lower than controls on the SET-CI subscale. For SET-EA and SET-IA, the most pronounced differences were observed between the AN and GP groups. Additional comparisons across EQ and TAS-20 subscales—both significant and non-significant—are reported in **Table 2**.

**Table 2:** Kruskal–Wallis test results and Dunn’s *post hoc* comparisons between groups.

Variable	H (2)	p-value	*Post hoc*
SET-CI	23.65	<.001 *	AN < GP (p <.001) bAN < GP (p <.001)
SET-EA	20.31	<.001 *	AN < GP (p <.001) bAN < GP (p = .038)
SET-IA	13.22	.001 *	AN < GP (p = .001) bAN < GP (p = .042)
GSET	33.79	<.001 *	bAN < AN (p = 0.038) AN < GP (p < 0.001) bAN < GP (p = 0.001)
RMET	15.36	<.001 *	AN < GP (p <.001) bAN < GP (p = .034)
EQ-CE	0.45	.798	
EQ-EE	6.49	.039 *	GP < AN (p = .035)
EQ-SS	22.48	<.001 *	AN < GP (p = .041) AN < GP (p <.001)
Total EQ	3.55	.170	
TAS-DIF	33.49	<.001 *	GP < AN (p < 0.001) bAN < AN (p = 0.001) GP < bAN (p = 0.029)
TAS-DDF	18.76	<.001 *	GP < AN (p < 0.001) bAN < AN (p = 0.045) GP < bAN (p = 0.036)
TAS-EOT	5.15	.076	
Total TAS-20	25.97	<.001 *	GP < AN (p < 0.001) bAN < AN (p = 0.042) GP < bAN (p = 0.025)

H(2): Kruskal–Wallis H test with 2 degrees of freedom. Dunn’s *post hoc* tests with Bonferroni correction applied. AN, patients with anorexia nervosa; bAN, brothers of AN patients; GP, general population controls; SET, story-based empathy test; CI, causal inference; EA, emotional states; IA, identifying intentions; GSET, Global score of SET; EQ, empathy quotient; CE, cognitive empathy; EE, emotional empathy; SS, social skills; TAS-20, Toronto alexithymia scale; DIF, difficulty identifying feeling; DDF, difficulty describing feelings; EOT, externally oriented thinking; GSET, Global score of SET. *Denotes statistically significant differences (p <.05).

To assess multicollinearity among predictors in the logistic regression model, VIF values were calculated for all variables. All VIFs ranged from 1.08 to 2.69, well below the commonly accepted thresholds of 5 or 10, indicating no concerning collinearity. The highest VIF values were observed for the two alexithymia subscales—Difficulty Identifying Feelings (VIF = 2.64) and Difficulty Describing Feelings (VIF = 2.69)—which is expected given their conceptual and statistical overlap. However, these values remain within acceptable limits, supporting the inclusion of all eight predictors in the regression model without risk of distortion due to multicollinearity.

A binary logistic regression was conducted to assess whether specific empathy- and ToM-related variables significantly predicted group membership (bAN vs. GP). The model included eight predictors: RMET, SET-IA, SET-CI, SET-EA, EQ-EE, EQ-SS, TAS-DDF, and TAS-DIF. The overall model was statistically significant, χ²(8, N = 62) = 29.38, p <.001, explaining 50.3% of the variance (Nagelkerke R²). The only variable significantly associated with group membership was SET-CI, with lower SET-CI scores increasing the likelihood of belonging to the bAN group (B = 1.976, SE = 0.694, Wald = 8.112, p = .004, OR = 7.213, 95% CI [1.852, 28.097]). All other predictors, including RMET, EQ subscales, and TAS-20 subscales, did not reach significance. See **Table 3** for details.

**Table 3:** Binary logistic regression predicting group membership (bAN vs. GP) from cognitive and emotional variables.

Predictor	B	SE	Wald	p	OR	95% CI for OR
RMET	-0.042	0.216	0.038	.845	0.959	[0.628, 1.463]
SET-IA	-0.222	0.566	0.154	.695	0.801	[0.264, 2.426]
SET-CI	**1.976**	**0.694**	**8.112**	**.004**	**7.213**	**[1.852, 28.097]**
SET-EA	0.713	0.622	1.312	.252	2.040	[0.602, 6.905]
EQ-EE	-0.220	0.230	0.920	.337	0.802	[0.511, 1.259]
EQ-SS	0.097	0.169	0.330	.566	1.102	[0.791, 1.534]
TAS-DDF	-0.104	0.125	0.698	.403	0.901	[0.705, 1.151]
TAS-DIF	-0.035	0.075	0.224	.636	0.965	[0.834, 1.117]

The model was statistically significant, χ²(8, N = 62) = 29.38, p <.001, explaining 50.3% of the variance (Nagelkerke R²) and correctly classifying 80.6% of cases. AUC = 0.876. CI, Causal Inference; RMET, Reading the Mind in the Eyes Test; SET: story-based empathy test; IA, identifying intentions; CI, causal inference; EA, emotional states; EQ, empathy quotient; EE, emotional empathy; SS, social skills; TAS, Toronto alexithymia scale; DDF, difficulty describing feelings; DIF, difficulty identifying feelings. Boldface indicates statistical significance (p <.05).

A sensitivity analysis was conducted by re-running the logistic regression model without the SET-CI predictor. The revised model remained statistically significant, χ²(7, N = 62) = 30.45, p <.001, explaining 35.4% of the variance and correctly classifying 80.6% of cases. In this model, TAS-DIF was the only significant predictor of group membership (B = 0.218, SE = 0.076, Wald = 8.12, p = .004, OR = 1.243, 95% CI [1.071, 1.444]). All other predictors remained non-significant.

## Discussion

This study explored social-cognitive and emotional processing in male siblings of individuals with anorexia nervosa, a population rarely investigated in the context of familial vulnerability to eating disorders. The findings indicate that bAN participants showed significantly lower performance than general population controls on several tasks assessing ToM and inferential reasoning. Additionally, they reported higher levels of alexithymia, particularly in difficulties identifying and describing feelings, reinforcing the view that emotional processing differences may not be limited to individuals with clinical eating disorders.

Among these variables, causal inference emerged as the most robust discriminator of group membership, with the strongest effect size and a significant predictive role in the logistic regression. While originally conceptualized as a control condition in SET paradigms (30), causal inference may engage domain-general reasoning and integrative functions—such as weighing contextual cues, mental state attribution, and sequencing of events—that are crucial for adaptive social cognition (32). These findings echo prior literature linking causal inference performance to broader neurocognitive vulnerabilities in conditions such as autism spectrum disorder and schizophrenia (33–35), and suggest that it may warrant greater attention as a potentially sensitive marker in transdiagnostic risk models.

The observation of group differences across other performance-based ToM tasks but not in all self-reported empathy questionnaires could underscore an important dissociation between subjective and objective social-cognitive measures. This may reflect known limitations of introspective access to empathic ability, particularly in populations with elevated alexithymia (36). Moreover, sex-related variability in affective reporting, with males often underreporting emotional difficulties or scoring lower on affective empathy (15), could attenuate group differences in questionnaire-based assessments. This distinction highlights the importance of combining multiple assessment modalities in the study of social cognition.

Notably, the presence of specific cognitive impairments—particularly in theory of mind and causal inference abilities—in siblings without a history of eating disorders echoes patterns commonly observed in individuals with AN (17, 18), and adds to the literature on potential familial or trait-like markers of vulnerability to the disorder. While previous studies have identified intermediate cognitive-affective profiles in unaffected sisters (22, 24–26), investigations of male siblings remain scarce. These results suggest that familial liability may manifest through subtle neurocognitive signatures—particularly those involving reasoning and emotion integration—even in males who do not develop overt psychopathology. However, the cross-sectional nature of the study precludes conclusions about causality or developmental sequence, and longitudinal designs will be necessary to determine whether these traits predict future clinical outcomes.

Another consideration relates to the possibility of compensatory mechanisms. The relatively preserved scores on some self-report measures could reflect successful cognitive adaptation in unaffected siblings, despite underlying differences in processing styles. This is consistent with resilience literature suggesting that individuals at risk for psychiatric disorders may develop alternative strategies to maintain social or emotional functioning (37). Alternatively, the findings may reflect latent vulnerabilities that require interaction with environmental or stress-related triggers to become clinically relevant.

In an exploratory sensitivity analysis excluding SET-CI from the regression model, TAS-DIF emerged as a statistically significant predictor of group membership. This result may suggest that, when causal inference is not accounted for, difficulties in emotional awareness contribute more prominently to the differentiation between bAN and control participants. Although SET-CI remained the only significant predictor in the full model, this secondary finding highlights the potential relevance of alexithymic traits in the broader context of familial vulnerability to AN. Given the limited sample size and the exploratory nature of this analysis, these results should be interpreted with caution. Nevertheless, the finding are consistent with previous research linking alexithymia to emotional dysregulation in AN (13) and aligns with the view that alexithymia may not only co-occur with AN but could also represent a transdiagnostic vulnerability marker (38, 39), even in the absence of overt pathology. Finally, these results contribute to the growing recognition that eating disorders in males remain underdiagnosed and under-researched (2). While the clinical presentation of AN may differ by sex, the presence of similar cognitive-affective vulnerabilities in male relatives reinforces the need to move beyond gendered assumptions in both research and prevention. Identifying subtle familial markers—such as impairments in causal inference or emotional processing—may support the development of more targeted early detection strategies, particularly in populations that do not typically present with classical symptom profiles.

### Clinical implication

The present findings offer relevant insights for clinical practice, particularly in the context of early identification and prevention strategies within families affected by AN. The presence of cognitive and emotional differences in unaffected male siblings—specifically deficits in causal reasoning and elevated alexithymic traits—suggests that subtle vulnerabilities may be detectable even in the absence of manifest psychopathology. These traits, often associated with poor intersubjective functioning and emotion regulation difficulties in AN patients (40, 41), may compromise resilience or increase sensitivity to environmental stressors over time.

From a clinical standpoint, the assessment of reasoning and emotional processing styles in siblings of individuals with AN may provide valuable information for identifying at-risk individuals. Incorporating brief performance-based tasks (e.g., causal inference tasks or theory of mind assessments) alongside validated self-report tools for alexithymia could enhance routine family evaluations, particularly in adolescent or early adulthood stages.

Importantly, these findings support the extension of preventive strategies to male family members, who are often underrepresented in both research and clinical interventions. Psychoeducational programs targeting families could benefit from including components on emotional awareness, perspective-taking, and flexible reasoning styles, not only for the affected patient but also for siblings and caregivers.

Furthermore, interventions previously shown to improve cognitive-affective functioning in AN patients—such as cognitive remediation therapy (CRT) or social-cognitive training (19, 42)—might be adapted and piloted in at-risk siblings, especially those displaying marked emotional dysregulation or rigidity. These findings suggest that specific cognitive-emotional traits, such as difficulties in causal inference, may be present in individuals without a formal psychiatric diagnosis and could reflect underlying vulnerability markers relevant beyond categorical diagnostic boundaries.

### Limitations and future directions

Several limitations warrant consideration. The relatively small and demographically homogeneous sample may constrain the generalizability of findings, and replication in larger, more diverse cohorts is necessary. While recruitment of male siblings of individuals with AN presents substantial challenges, expanding sample size is essential for improving statistical power and refining the characterization of familial cognitive-emotional traits.

The cross-sectional nature of the study also limits interpretation of the temporal and causal nature of the observed associations. Longitudinal designs are crucial to determine whether cognitive-emotional features identified in male siblings predict later psychopathology or function as stable endophenotypic markers. In particular, future work should clarify whether impairments in causal inference represent trait-like vulnerabilities or reflect compensatory strategies shaped by shared familial dynamics.

Furthermore, although the causal inference subscale was not originally developed to assess social reasoning, its predictive role in group membership suggests a need to reevaluate its interpretive scope. Future studies should include more differentiated instruments capable of disentangling domain-general inferential deficits from social-cognitive impairments.

Finally, research would benefit from the inclusion of other family members—such as parents and unaffected sisters—to better delineate familial versus gender-specific effects. Investigating the potential contribution of environmental, epigenetic, and neurobiological factors may provide further insight into the intergenerational transmission of risk.

## Conclusions

This study contributes to an evolving understanding of the cognitive-affective architecture underlying familial vulnerability to AN. The presence of inferential reasoning impairments and elevated alexithymic traits in unaffected male siblings suggests that these individuals may express subclinical manifestations of traits associated with AN. These findings lend support to the conceptualization of cognitive-affective dimensions as transdiagnostic markers, extending beyond symptomatic expression and diagnostic boundaries.

Rather than focusing solely on global empathy or traditional markers of social cognition, these results emphasize the relevance of more nuanced reasoning processes—such as causal inference—in shaping vulnerability to psychopathology. Future research should adopt longitudinal and multi-informant designs to better understand the developmental trajectories of these traits, as well as their interaction with gender, environment, and neurocognitive functioning. By identifying specific cognitive-emotional profiles in at-risk individuals, we move closer to developing precision-based approaches to prevention and early intervention in eating disorders.

## Data Availability

The raw data supporting the conclusions of this article will be made available by the authors, without undue reservation.
